# A scavenging system against internal pathogens promoted by the circulating protein apoptosis inhibitor of macrophage (AIM)

**DOI:** 10.1007/s00281-018-0717-6

**Published:** 2018-10-11

**Authors:** Satoko Arai, Toru Miyazaki

**Affiliations:** 1 Laboratory of Molecular Biomedicine for Pathogenesis, Center for Disease Biology and Integrative Medicine, Faculty of Medicine, The University of Tokyo, 7-3-1 Hongo, Bunkyo-ku, Tokyo, 113-0033 Japan; 2 AMED-CREST, Japan Agency for Medical Research and Development, Tokyo, Japan; 3 Max Planck-The University of Tokyo Center for Integrative Inflammology, Tokyo, Japan

**Keywords:** Apoptosis inhibitor of macrophage, Non-professional phagocyte, Acute kidney injury, Scavenger receptor, Macrophage, Phagocytosis

## Abstract

An internal system designed to ward off and remove unnecessary or hazardous materials is intrinsic to animals. In addition to exogenous pathogens, a number of self-molecules, such as apoptotic or necrotic dead cells, their debris, and the oxides or peroxides of their cellular components, are recognized as extraneous substances. It is essential to eliminate these internal pathogens as quickly as possible because their accumulation can cause chronic inflammation as well as autoimmune responses, possibly leading to onset or progression of certain diseases. Apoptosis inhibitor of macrophage (AIM, also called CD5L) is a circulating protein that is a member of the scavenger receptor cysteine-rich superfamily, and we recently found that during acute kidney injury, AIM associates with intraluminal dead cell debris accumulated in renal proximal tubules and enhances clearance of luminal obstructions, thereby facilitating repair. Thus, AIM acts as a marker for phagocytes so that they can efficiently recognize and engulf the debris as their targets. In this chapter, we give an overview of the professional and non-professional phagocytes, and how soluble scavenging molecules such as AIM contribute to improvement of diseases by stimulating phagocytic activity.

## Professional and non-professional phagocytes

**Fig. 1:**
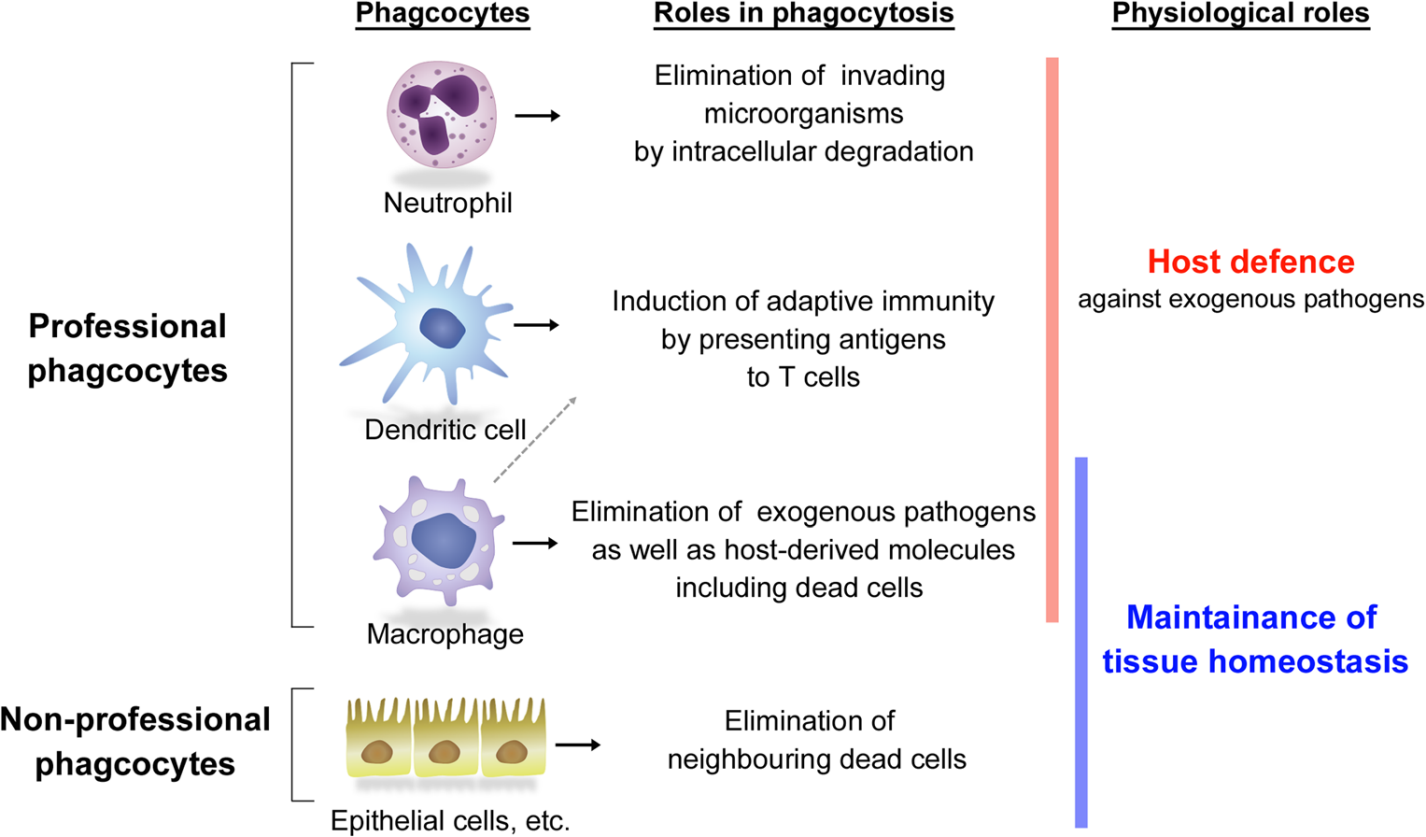
Scheme of representative phagocytes and their roles

Phagocytosis of both exogenous pathogens and self-derived unnecessary substances is induced when the targets are recognized by the receptors expressed on the surface of phagocytes, which triggers activation of the subsequent phagocytic process. It is well known that several leukocytes, such as neutrophils, dendritic cells (DCs), and macrophages, are “professional phagocytes” that express specific receptors abundantly on their cell surface to capture targets and harbor highly developed lysosomes to efficiently digest and decompose the internalized material (Fig. 1). Neutrophils protect the host by quickly migrating into the sites affected by exogenous pathogens to eliminate them via phagocytosis, while DCs can induce an advanced immune system that is provided with the antigen specificity through presenting antigenic peptides, which are created from the engulfed targets, by the major histocompatibility complex (MHC) to T cells. Phagocytosis executed by these two phagocytes principally contributes to host defense mechanisms against infection.

Macrophages, which are distributed in every tissue, had been long thought to be differentiated from bone marrow-derived monocytes. However, accumulated evidence has indicated that tissue-resident macrophages originate not from adult bone marrow hematopoiesis, but from the primitive hematopoiesis that is carried out either in the yolk sac or in the embryonic liver [1–3]. Although there is a certain population that is derived from monocytes mainly involved in inflammation, this subset has clearly been distinguished from that of tissue-resident macrophages over the past several decades, such as microglia in the central nervous system, Langerhans cells in skin, liver Kupffer cells, lung alveolar macrophages, and splenic red pulp macrophages. These tissue-resident macrophages have phagocytic capacity in common, whereas each subset has its own functions that are provided by the tissue-specific maturation process as well as the characteristic gene expression profiles brought about by particular environmental factors, and thus contributes to maintenance of the functionality and the structure of the tissue in which the macrophage resides [4].

In addition to these professional phagocytes, which are all descendants of hematopoietic stem cells, it has been reported that several non-leukocyte-lineage cells such as epithelial cells also play phagocytic roles in a variety of tissues in response to environmental necessities. These are called “non-professional phagocytes” (Fig. 1). Rapid engulfment of dead cells, which died either by aging or by tissue damage, by neighboring cells prevents a harmful explosion of inflammatogenic intracellular contents and promotes efficient reconstruction of damaged tissues, thereby maintaining tissue homeostasis. For example, in spermatogenesis, half of the spermatogenic cells undergo apoptosis during meiosis. Sertoli cells, a type of sustentacular cell of the testicles, rapidly engulf those apoptotic cells, supporting efficient sperm production [5, 6]. Also, in mammary glands, along with post-lactational involution after weaning, the vast majority of mammary epithelium dies in apoptosis within a short period. These substantial numbers of apoptotic cells are removed by neighboring mammary epithelial cells, and this process is required for remodeling of the tissue and subsequent lactation [7, 8]. Furthermore, a continuous phagocytosis by non-professional phagocytes also takes place for clearance of the released cellular components. In retinal photoreceptor cells, outer segments, which consist of stacked individual disk membranes that absorb photons, are constantly generated from the basal end and simultaneously shed from the distal end, thus maintaining a constant length. The released mature outer segments are swiftly eliminated by being engulfed by the retinal pigment epithelium, which is essential for retinal health [9, 10]. In any case, the phagocytic activities performed by the non-professional phagocytes contribute to the physiological tissue turnover or remodeling, leading to maintenance of the tissue homeostasis.

Besides its involvement in tissue turnover, the phagocytic removal of dead cells plays an indispensable role, especially in cases of acute tissue injury. Nakaya et al. reported that dead cells generated following myocardial infarction (MI) were efficiently engulfed by cardiac myofibroblasts, which suppressed the release of toxic intracellular contents from the dead cells that induce secondary cell death and inflammatory responses, thus preventing the expansion of the damaged area [11].

Similarly, it has been reported that during acute kidney injury (AKI), the surviving renal proximal tubular epithelial cells engulf the intraluminal dead cell debris that is generated by necrotic death of the proximal tubular epithelial cells, thereby promoting recovery [12, 13]. These results suggest that the clearance of dead cells performed by the neighboring cells, including both professional and non-professional phagocytes, is a fundamental mechanism to minimize damage and facilitate recovery.

## Receptors and ligands participating in phagocytosis

Recognition of ligands by receptors is the first step to internalizing the targets by the phagocyte. Therefore, specific molecular markers, including certain characteristic molecules or specific structures that can convey “I am a target” to phagocytes, should be provided to ligands for initiation of phagocytosis, while phagocytes should be equipped with receptors that can recognize those signals.

There are several types of receptors that induce phagocytosis, and they are basically divided into opsonin-dependent or opsonin-independent [14]. The former are mostly Fc receptors expressed on many immune cells that recognize Fc regions of immunoglobulins. In Fc receptor-mediated phagocytosis, pathogens such as bacteria are opsonized by specific antibodies and internalized via Fc receptors; therefore, adaptive immunity can maximize the activity of Fc receptor-dependent phagocytosis. On the other hand, Fc receptor-independent phagocytosis is more primitive since receptors do not need intermediation of specific antibodies but can directly capture pathogens.

**Fig. 2:**
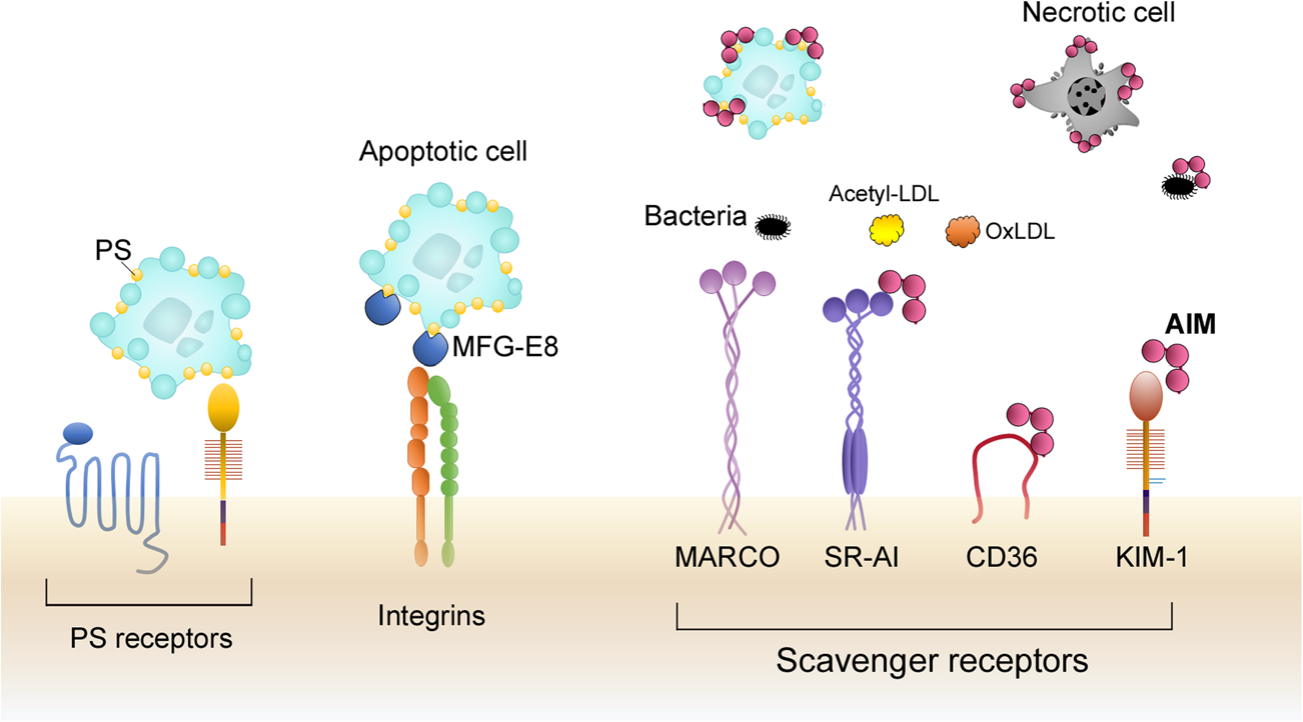
Scheme of receptors that induce phagocytosis and involvement of AIM. (*Left*) Phagocytosis of apoptotic cells is performed via receptors that recognize PS as well as soluble MFG-E8 protein that binds to both PS and a specific integrin expressed by phagocytes. (*Right*) A broad variety of scavenger receptors can recognize bacterial PAMPs or modified self-molecules including modified LDL as well as dead cells. AIM is recognized by a variety of scavenger receptors including CD36 and KIM-1 and is endocytosed by phagocytes together with the molecules (or dead cells) that are bound to AIM. The AIM protein itself also has a pattern recognition property like other scavenger receptors to bind to dead cells or PAMPs

The pattern recognition receptor (PRR) is one of the most well-known opsonin-independent receptors that recognizes pathogen-associated molecular patterns (PAMPs), including lipopolysaccharide (LPS) from Gram-negative bacteria, lipoteichoic acid (LTA) and peptidoglycan from Gram-positive bacteria, and β-glucans and mannans from yeasts [15]. Among PRRs, Toll-like receptors (TLRs) activate NF-κB signaling cascades to produce inflammatory cytokines, whereas a group of scavenger receptors including scavenger receptor-A (SR-A), MARCO, and CD36 can induce phagocytic activity (Fig. 2). Interestingly, while most TLRs contain extracellular leucine-rich repeats (LRRs) that recognize PAMPs and an intracellular Toll/interleukin-1 receptor (TIR) domain that activates a common signaling cascade [16, 17], few structural features are conserved among the scavenger receptors, while they have many functional similarities [18]. It is well known that the scavenger receptors not only contribute to host defense through promoting phagocytosis of microbial pathogens but also induce phagocytosis of host-derived molecules such as negatively charged molecules including modified lipoproteins (e.g., acetyl-LDL and oxidized-LDL), or advanced glycation end products [19]. Damage-associated molecular patterns (DAMPs) such as high-mobility-group box 1, peroxiredoxins, S100A8, and S100A9, which are released from dead cells, are also ligands for scavenger receptors [20]. It was reported that during ischemic cerebral stroke in mice, the clearance of DAMPs was largely mediated by the class A scavenger receptor MSR1, which attenuates inflammatory signals promoted by DAMPs, improving the cerebral pathology [21].

An important phagocytic system also exists principally for the elimination of apoptotic cells. Phosphatidylserine (PS), which is flipped from the inner leaflet of the plasma membrane to the extracellular surface of the cell under apoptosis, is one of the most famous “eat me” signals that induce phagocytes to engulf them. There are several receptors that directly recognize PS, including BAI1, T cell immunoglobulin and mucin domain 4 (TIM-4), stabilin 2, and kidney injury molecule-1 (KIM-1, also known as TIM-1) [22, 23]. The scavenger receptors SR-AI/II and MARCO also recognize and engulf apoptotic cells [18, 19, 23, 24].

Intriguingly, in addition to the direct ligand-receptor system, some soluble proteins support phagocytosis by bridging the targets and the receptors. Milk fat globule-epidermal growth factor 8 (MFG-E8) is a secreted molecule that binds to both PS exposed on apoptotic cells and the integrin receptors α_v_β3–5 expressed on phagocytes, thereby promoting removal of apoptotic cells (Fig. 2) [11, 25]. In the germinal centers in spleens and lymph nodes, MFG-E8 plays a central role in the elimination of the apoptotic B cells and suppresses autoimmune diseases [25]. MFG-E8 is also involved in the clearance of dead cells by cardiac myofibroblasts in the course of recovery during MI [11]. Hence, the bridging molecules that link the targets and the phagocytes appear to be important factors that constitute the effective phagocytic systems. Recently, we found that during AKI, the macrophage-derived circulating protein AIM enhances the phagocytic activity of the renal proximal tubular epithelial cells by attaching to the dead cell debris within the proximal tubules and thus promotes recovery from AKI [13]. In the next section, the characteristics of AIM as a soluble scavenger protein and the intriguing regulation of AIM’s contribution to disease repair are presented.

## AIM, a soluble scavenger protein

**Fig. 3:**
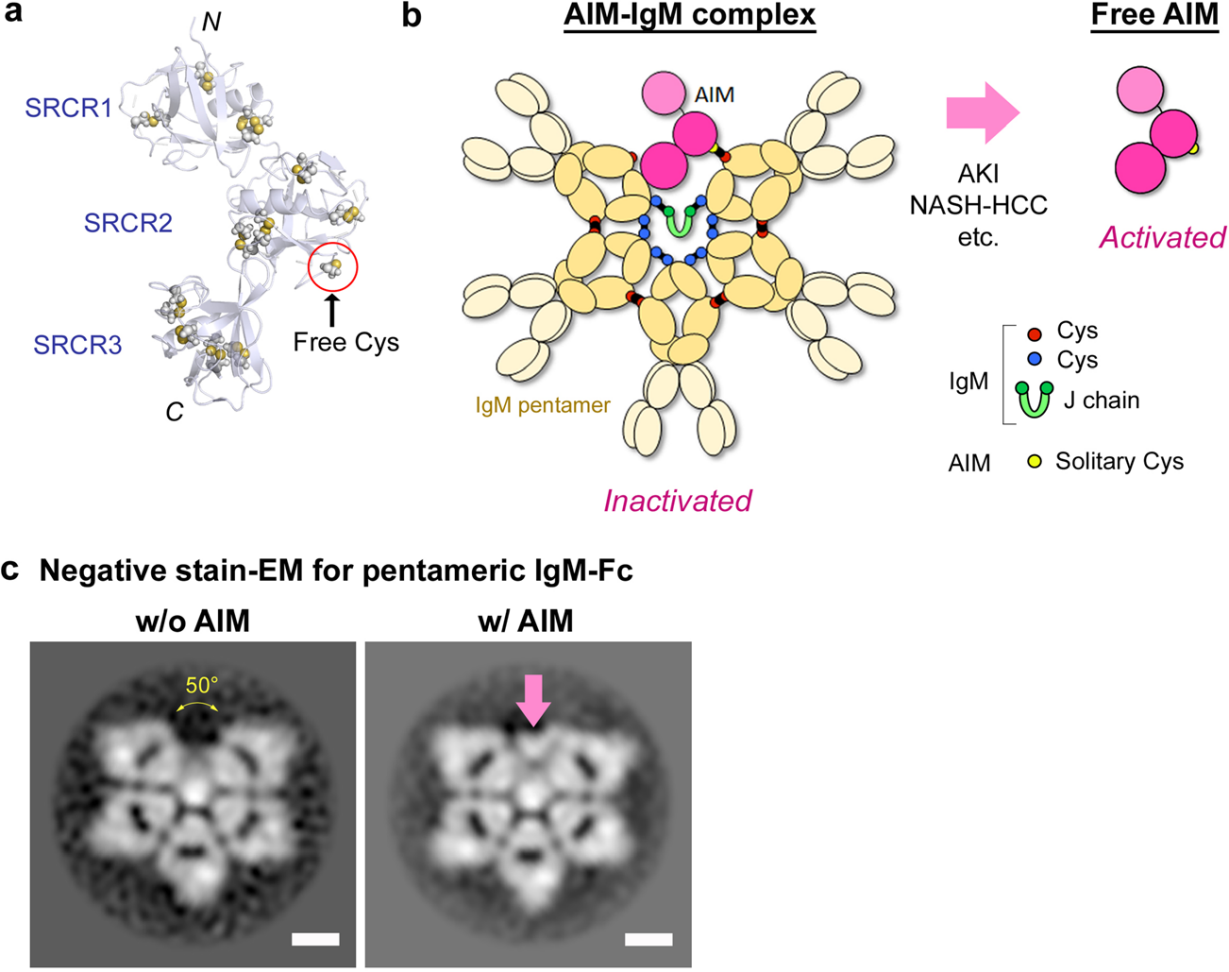
The structure of AIM and AIM-IgM complex. **a** Homology-based structural model of mouse AIM. Cysteine residues are indicated by spheres. The solitary cysteine residue that does not form an internal disulfide bond but is utilized for formation of a disulfide bond with IgM is located in the second SRCR domain. Other cysteine residues form internal disulfide bonds within each domain. The IgM-free AIM can translocate from blood to urine. **b** Schematic structural model of the AIM-IgM complex and IgM-free AIM. Our recent data obtained by single-particle negative-stain electron microscopy combined with analysis of reference-free 2D class averages (presented  in c)  show that the bona fide structure of the IgM pentamer is an asymmetric pentagon containing one large gap and that one AIM fits into the gap, cross-bridging two IgM Fc domains that form the edges of the gap through a disulfide bond on one side and a charge-based interaction on the other. Association with IgM prevents AIM from being excreted into urine due to the large molecular size (approx. 1000 kDa), thereby maintaining high serum levels of AIM; however, IgM-bound AIM is functionally inactivated. Dissociation is induced during AKI or in other specific diseases including non-alcoholic steatohepatitis (NASH)-induced hepatocellular carcinoma [45]. Red circles: the cysteine residues of IgM, which contribute to the assembly of IgM monomers and the association with AIM. Blue circles: the cysteine residues of IgM, which link both the tail regions of IgM monomers and joining (J) chain. J chain: small polypeptides that is necessary for the proper formation of pentameric IgM. Yellow circle: the solitary cysteine residue of AIM that forms disulfide bond with IgM. **c** Images of mouse IgM Fc pentamer without (left) or with (right) AIM obtained by negative-stain electron microscopy. AIM is indicated by arrow. Bars: 5 μm. Data are modified from a previously published report [31]

**Fig. 4:**
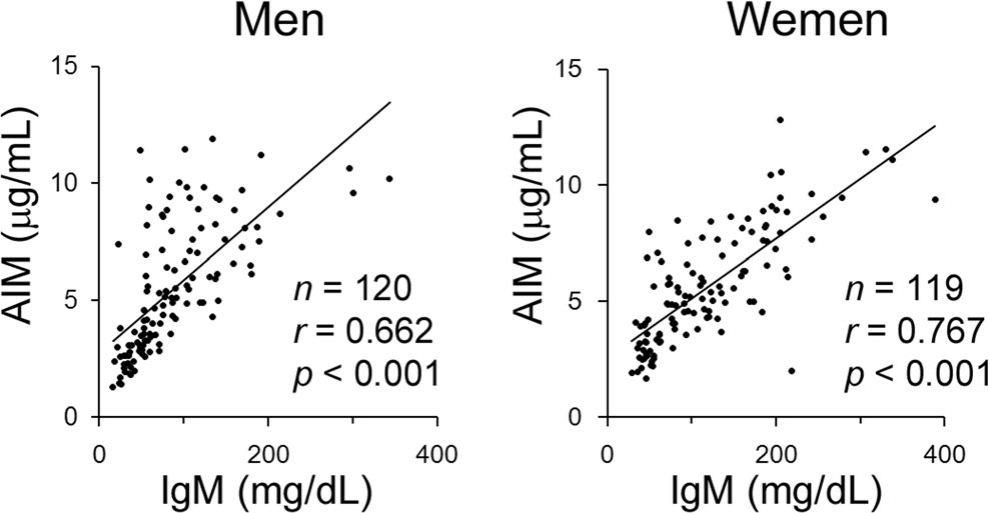
The correlation between serum levels of AIM and IgM in healthy human individuals. AIM and IgM levels from 20 healthy individuals from each generation (20s to 70s) are presented. Data are modified from a previously published report [32]

AIM, also known as CD5L, is a soluble protein of approximately 40 kDa (Fig. 3a), mainly produced by tissue-resident macrophages, including liver Kupffer cells and peritoneal macrophages, via the transcriptional activation of nuclear receptor liver X receptor/retinoid X receptor (LXR/RXR) heterodimers and/or the transcription factor MafB [26–28]. AIM belongs to the scavenger receptor cysteine-rich (SRCR) superfamily, which shares a highly conserved cysteine-rich domain of approximately 100 amino acids called the SRCR domain, and consists of three tandem-repeat SRCR domains. AIM is broadly conserved in mammals, including human, mouse, dog, and cat. As far as we are aware, human and mouse AIM proteins are functionally equivalent. In blood, AIM associates with pentameric immunoglobulin M (IgM), most likely in the ratio of one to one, and stably circulates without renal excretion (Fig. 3b) [29–31]. Recently, we discovered that the structure of the IgM pentamer is that of an asymmetric pentagon containing one large gap and is markedly different from the conventional symmetric pentagon model (Fig. 3b and c). Moreover, we observed that a single AIM fits into the gap of the IgM pentamer as if the cavity were specifically customized for AIM, and that the second and the third SRCR domains are important for association with IgM, facilitating a disulfide bond and a surface-charge interaction with the Fc domain of IgM, respectively (Fig. 3b) [31]. Because pentameric IgM is approximately 1000 kDa, the IgM-associated AIM cannot be filtrated into urine because of the size selectivity of the glomerular barrier. Therefore, the serum level of AIM is highly dependent on that of IgM, being maintained at relatively high concentration in humans and mice (approximately 5 and 3 μg/mL, respectively) in steady-state conditions (Fig. 4) [32, 33]. Interestingly, AIM levels are relatively higher in women (6.06 ± 2.09 μg/mL in women, 4.99 ± 1.76 μg/mL in men), and highest in both men and women in their 20s and decreased with age [32].

Similar to other SRCR-SF members such as MARCO or SR-AI, AIM exhibits pattern recognition. For example, AIM binds to Gram-negative and Gram-positive bacteria via binding to LPS, LTA, or peptidoglycan [18, 34], as well as host-derived dead cells [13]. Interestingly, within the cytosol, AIM binds to a fatty acid synthase, thereby suppressing its enzymatic activity [35, 36]. However, these binding capacities are veiled when AIM is associated with IgM [13, 31]. More interestingly, AIM itself is recognized by multiple scavenger receptors such as CD36 or KIM-1, which is strongly expressed by injured proximal tubular epithelial cells, thereby being endocytosed by the cells expressing those receptors (Fig. 2) [13, 35, 37]. It is reported that AIM is incorporated by various types of cells, including hepatocytes, adipocytes, and injured proximal tubular epithelial cells, as well as some subsets of resident macrophages such as peritoneal macrophages or Kupffer cells [13, 35, 38]. When AIM is engulfed by phagocytes, the molecules that are bound to AIM are also engulfed. Accordingly, AIM acts as a “search and destroy” weapon that is discharged from a macrophage and circulates throughout the body to remove cellular debris. Interestingly, this function is maximized when AIM is dissociated from IgM. IgM association restricts not only target-binding capacity but also the ability to reach particular regions of the body, especially during AKI [13].

## The clearance of intraluminal dead cell debris mediated by AIM during AKI

**Fig. 5:**
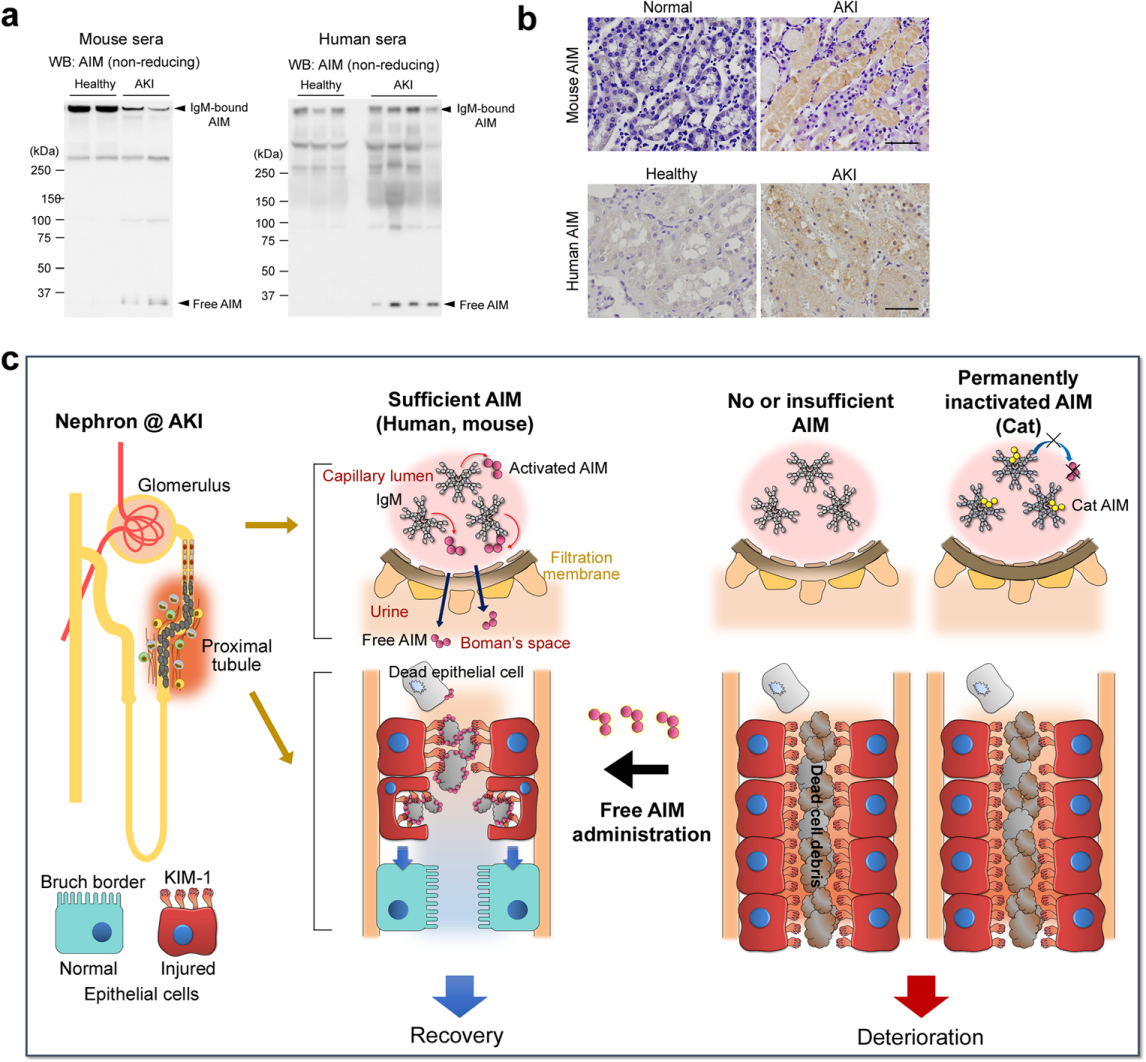
Involvement of AIM in AKI pathogenesis. **a** Western blotting under non-reducing conditions for AIM *in sera* from control and AKI-induced wild-type mice (*n* = 2 each, *left*), and healthy individuals (*n* = 3) and AKI patients (*n* = 5). IgM-bound AIM and IgM-free AIM are indicated by arrows. IgM-free AIM is detected only in AKI conditions, whereas IgM-bound AIM is decreased in AKI in mice. **b** Immunohistochemical staining for AIM of the kidney specimens of healthy and AKI mice (upper panels), as well as a healthy individual and an AKI patient (lower panels). Signals were visualized by horseradish peroxidase/3-diaminobenzidine. Scale bars: 50 μm. Data are modified from a previously published report [13]. **c** The schematic structure of a nephron and a schema of AIM states and proximal tubules during AKI in wild-type mice, humans, AIM-deficient mice, and cats are depicted. In wild-type mice and most humans, sufficient IgM-free AIM typically dissociates from the IgM pentamer during AKI and is filtrated through glomeruli into the urine, accumulating on the intraluminal dead cell debris and thereby enhancing its clearance by injured proximal tubular epithelial cells (indicated by red cells) via KIM-1, leading to the regeneration of epithelial cells (indicated by blue cells) and AKI recovery. In the absence of AIM (for example, AIM-deficient mice), this debris removal is deficient. In cats, due to the high affinity between AIM and IgM, AIM is unable to dissociate from IgM during AKI, abolishing its excretion in urine; the intraluminal debris therefore cannot be removed efficiently. rAIM administration could be therapeutically applied for AKI treatment

AKI is characterized by abrupt impairment of kidney filtration function caused by various insults and is one of the diseases in which AIM plays an important role for recovery. Renal ischemia-reperfusion (IR), as well as cardiac or systemic IR, which induces production of free radicals, causes tissue injury especially in renal proximal tubules, resulting in cell death of renal proximal tubular epithelial cells followed by intraluminal obstruction. Persistent accumulation of dead cell debris in the lumen induces not only augmentation of intraluminal pressure resulting in a decrease in glomerular function, but also inflammation along with fibrosis, leading to prolonged tissue injury and decreased renal function. Therefore, rapid resolution of dead cell occlusion is indispensable for recovery of the epithelium and reconstruction of functional tissue. As mentioned above, AIM does not reach the lumen under normal conditions, as IgM-associated AIM cannot pass through the glomerulus. However, we found that AIM is systemically dissociated from IgM during AKI (although the mechanism is not yet elucidated) (Fig. 5a) and translocates to the urine, thereby accumulating on the intratubular debris [13] (Fig. 5b). Interestingly, there are no professional phagocytes such as macrophages within the lumen, but non-professional phagocytes, in this case injured but surviving renal proximal tubular epithelial cells, play a central role in debris clearance by highly expressing KIM-1 on the luminal side [12, 37]. The AIM protein attached to the cellular debris binds to KIM-1 and enhances the phagocytic removal of the debris by the epithelial cells, thus contributing to kidney tissue repair (Fig. 5c) [13]. Although it had already been reported that KIM-1 directly recognizes PS and engulfs apoptotic cells [36, 38], we demonstrated that AIM-dependent dead cell clearance is crucial [13]. When mice were subjected to IR-induced AKI, AIM-deficient mice exhibited abrogated debris clearance and persistent renal inflammation, resulting in higher mortality than in wild-type mice due to progressive renal dysfunction [13]. Treatment of mice with IR-induced AKI in AIM-deficient mice using recombinant AIM (rAIM) resulted in the removal of the debris, thereby ameliorating renal pathology (Fig. 5c) [13].

Additionally, we discovered that cats have a distinct feature in their AIM protein, which associates with IgM far more strongly than that of humans and mice [33]. The affinity between AIM and IgM is about 1000 times higher in cats than that in humans and mice according to the *K*_D_ values measured by surface plasmon resonance analysis [33]. Due to this unique characteristic, cat AIM never dissociates from IgM even during AKI; thus, AIM cannot reach the urine and AKI is not efficiently ameliorated even though blood AIM levels in cats are very high (approximately 20 μg/mL on average) (Fig. 5c) [33]. In other words, cat AIM is permanently inactivated. In fact, it is well known that cats are profoundly more susceptible to and more often die from renal failure than other animals. However, the exact reason for their susceptibility to renal disease, which has been one of the most pressing questions in veterinary medicine, has not been fully elucidated. We demonstrated that one of the possible reasons for the high frequency of renal diseases in cats was this permanent inactivation of AIM.

## Deficiency in the scavenging system and diseases

**Fig. 6:**
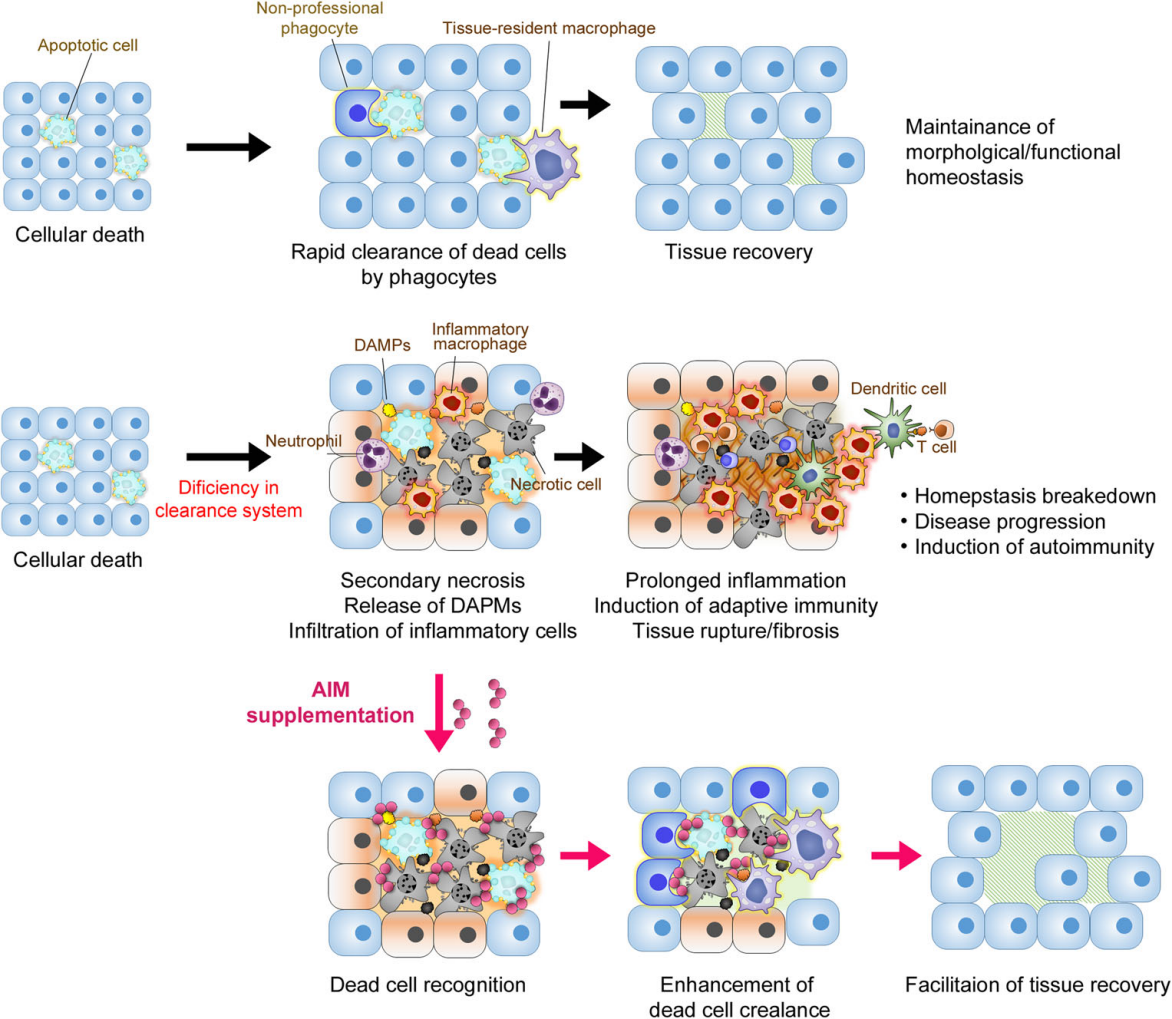
Schema for the deficiency in scavenging system and diseases. Typically, dead cells are rapidly removed by neighboring cells or by phagocytes such as macrophages, maintaining tissue homeostasis. In cases of deficiency of this removal system or massive injury, accumulation of dead cells causes prolonged inflammation, resulting in structural and functional tissue failure. Administration of rAIM may promote tissue recovery by enhancing clearance of dead cells and other dead cell-derived harmful molecules

As is the case for AKI, rapid clearance of dead cells by phagocytes is indispensable for maintenance of homeostasis in every tissue. Deficiency or insufficiency in this removal system may cause the following cascade: accumulation of dead cells induces inflammation, which enhances release of dead cell-derived materials including DAMPs, resulting in further prolongation of inflammation along with exacerbation of fibrosis, leading to structural and functional tissue failure (Fig. 6).

Recently, it was revealed that AIM-dependent dead cell removal is also important for recovery from fungus-induced peritoneal injury [39]. Fungal peritonitis is known to be a serious complication that occasionally occurs in patients on peritoneal dialysis (PD). Severe inflammation along with continuing cell death followed by infiltration of a number of neutrophils causes peritoneal membrane dysfunction, which necessitates discontinuation of PD. We found that AIM binds to dead cells in the peritoneum and enhances clearance, thereby contributing to recovery in a similar manner as in AKI. When mice were subjected to zymosan-induced peritonitis, AIM-deficient mice exhibited sustained peritoneal necrotic tissue with progressive inflammation as compared with wild-type mice [39]. Again, treatment of AIM-deficient mice with zymosan-induced peritonitis using rAIM resulted in the removal of the debris, thereby ameliorating peritoneal pathology [39]. Furthermore, when human patients on PD were analyzed, serum AIM levels were significantly lower in patients who had previously experienced peritonitis than in those without peritonitis [39], suggesting that AIM has a protective role to play against peritonitis in PD patients.

Other than AIM, as mentioned previously, MFG-E8, which bridges between apoptotic cells and phagocytes, is important for removal of dead cells that are produced in MI [11]. Upon MI, MFG-E8 is expressed by cardiac myofibroblasts mainly in the infarct and border areas, and supports engulfment of dead cells, resulting in promoting recovery. MFG-E8-deficient mice demonstrated the accumulation of dead cells after MI, resulting in exacerbated inflammation and thereby a substantial increase in mortality [11]. In addition, administration of MFG-E8 into infarcted hearts restored cardiac function and tissue structure [11].

## Other effects provided by engulfment of dead cells

In addition to elimination of the inflammatory “seed” by phagocytes, it is known that engulfment of apoptotic cells provides anti-inflammatory properties to phagocytes such as production of anti-inflammatory cytokines (e.g., TGF-β, IL-10) and decreased secretion of the proinflammatory cytokines (e.g., TNF-α, IL-1, and IL-12) [11, 40, 41], leading to suppression of further inflammatory and immune responses.

Furthermore, it is likely that efficient phagocytic removal of dead cells by non-professional phagocytes such as epithelial cells, which is observed both in normal tissue turnover and in some diseases such as AKI or MI, or even by macrophages, both of which do not present antigen as efficiently as DCs, also supports suppression of unnecessary adaptive immune responses that may cause autoimmunity, by depriving professional antigen-presenting cells of dead cell-derived autoantigens. For example, it was reported that KIM-1-mediated phagocytosis of apoptotic cells by renal proximal tubule cells down-modulates inflammation by reducing the proportion of effector T cells while increasing the proportion of regulatory T cells, through autophagic processing of engulfed materials to MHC class I and II, which induces a pro-tolerogenic T cell response [42] .

Indeed, it has been suggested that there is a significant relationship between development of autoimmune diseases and defects in eliminating dead cells. It was reported that defective phagocyte function resulting in insufficient dead cell clearance was observed in mice with systemic lupus erythematosus or with type I diabetes [43, 44].

## Perspectives for therapeutic application

It is intriguing that in many injury-associated diseases, including AKI, fungal peritonitis, or MI, soluble molecules such as AIM or MFG-E8 significantly participate in an efficient dead cell clearance in a similar fashion. It appears to be important and interesting that a common machinery is utilized for tissue recovery in different diseases as well as in different organs. More importantly, administration of these soluble proteins in such disease models could cure the diseases by promoting the phagocytic activity against dead cells [11, 13, 39], suggesting that these molecules can be used for therapeutic applications, not just for one specific disease but for several diseases (Fig. 6).

AIM may be the appropriate candidate. AIM abundantly circulates in almost every organ through the blood stream but is generally inactivated by the association with IgM, which in the meantime maintains AIM at high serum concentration by preventing renal excretion. This constant maintenance of high AIM levels by IgM association in healthy conditions is very important for quickly responding to incidents such as massive cell death in AKI. The balance of the two reciprocal states of AIM, between “stable but inactivated” and “activated,” is controlled by whether it is associated with or dissociated from pentameric IgM, although the factor(s) that turns on the activation/dissociation, which is observed during AKI, is not yet identified. In addition, our accumulating data have shown that AIM may recognize a broad spectrum of internal pathogens other than dead cells, including various toxins via its pattern recognition property (manuscript in preparation). Thus, we expect that AIM administration or artificial activation of AIM, for example by induction of release of AIM from IgM by small-molecule therapeutics, can be clinically applied for therapy of appropriate diseases such as AKI and fungal peritonitis, and other injury- or inflammation-associated diseases.
